# Exhaustion of CD8^pos^ central memory regulatory T cell differentiation is involved in renal allograft rejection

**DOI:** 10.3389/fimmu.2025.1532086

**Published:** 2025-01-24

**Authors:** Florian Kälble, Jonas Leonhard, Martin Zeier, Oliver Zivanovic, Matthias Schaier, Andrea Steinborn

**Affiliations:** ^1^ Department of Nephrology, University of Heidelberg, Heidelberg, Germany; ^2^ Department of Obstetrics and Gynecology, University of Heidelberg, Heidelberg, Germany

**Keywords:** kidney transplantation, immunosuppressive therapy, rejection, CD8^pos^ T cell differentiation, CD8^pos^ responder T cells, CD8^pos^ regulatory T cells, CD8^pos^ T cell exhaustion

## Abstract

**Background:**

The role of regulatory CD8^pos^ T cells (CD8^pos^ Tregs) and cytotoxic CD8^pos^ responder T cells (CD8^pos^ Tresps) in maintaining stable graft function in kidney transplant recipients (KTR) remains largely unclear. The pathogenesis of graft deterioration in case of rejection involves the exhaustive differentiation of both CD8^pos^ T cell subsets, but the causal mechanisms have not yet been identified.

**Methods:**

In this study, we separately investigated the differentiation of CD8^pos^Tregs/Tresps in 134 stable KTR with no evidence of renal graft rejection, in 41 KTR diagnosed with biopsy-confirmed rejection at enrolment and in 5 patients who were unremarkable at enrolment, but developed rejection within three years of enrolment. We were investigating whether changed differentiation of CCR7^pos^CD45RA^pos^CD31^pos^ recent thymic emigrant (RTE) cells via CD45RA^neg^CD31^pos^ memory (CD31^pos^ memory) cells (pathway 1), via direct proliferation (pathway 2), or via CCR7^pos^CD45RA^+^CD31^neg^ resting mature naïve (MN) cells (pathway 3) into CD45RA^neg^CD31^neg^ memory (CD31^neg^ memory) cells affects the CD8^pos^ Treg/Tresp ratio or identifies a CD8^pos^ Treg/Tresp subset that predicts or confirms renal allograft rejection.

**Results:**

We found that RTE Treg differentiation via pathway 1 was age-independently increased in KTR, who developed graft rejection during the follow-up period, leading to abundant MN Treg and central memory Treg (CM Treg) production and favoring a strongly increased CD8^pos^ Treg/Tresp ratio. In KTR with biopsy-confirmed rejection at the time of enrolment, an increased differentiation of RTE Tregs into CCR7^neg^CD45RA^pos^CD31^neg^ terminally differentiated effector memory (CD31^neg^ TEMRA Tregs) and CD31^pos^ memory Tregs was observed. CD31^neg^ memory Treg production was maintained by alternative differentiation of resting MN Tregs, resulting in increased effector memory Treg (EM Treg) production, while the CD8^pos^ Treg/Treg ratio was unaffected. An altered differentiation of CD8^pos^ Tresps was not observed, shifting the Treg/Tresp ratio in favor of Tregs.

**Conclusions:**

Our results show that exhaustive CD8^pos^ Treg differentiation into CM Tregs may lead to future rejection, with a shift towards EM Treg production and an accumulation of CD31^neg^ TEMRA Tregs in KTR with current rejection.

## Introduction

Kidney transplantation is the preferred therapy of patients with end-stage kidney failure since it reduces mortality and improves the quality of life compared to dialysis treatment (1, 2). Little is known about the mechanisms of regulatory T cells (Tregs) and responder T cells (Tresps) in patients with stable transplant kidney function compared to those who experience a deterioration in kidney function. Contrary to the well-studied regulatory CD4^pos^ T helper cells (CD4^pos^ Tregs), the role of regulatory CD8^pos^ cytotoxic T cells (CD8^pos^ Tregs) in maintaining tolerance to a transplanted kidney remains largely unclear. This lack of understanding is primarily due to the extremely low frequency of CD8^pos^ Treg cells, which can constitute as little as approximately 0.1% of CD8^pos^ T cells in mice and 0.4% in humans. Nonetheless, similar to CD4^pos^ Tregs, these cells have been found to co-express CD25 and FoxP3, display low levels of CD127, and exhibit heightened expression of activation or proliferation markers such as CTLA-4, ICOS, and Ki67 compared to CD8^pos^ cytotoxic effector T-cells (CD8^pos^ Tresps) (3). Although CD8^pos^FoxP3^pos^ T cells have been identified among single positive thymocytes (4), an exclusive thymic origin seems unlikely, as numerous studies have documented the generation of peripheral CD8^pos^FoxP3^pos^ T cells in the context of transplantation tolerance (5–7). These cells have been shown to exhibit potent class-I restricted suppression through various molecular mechanisms (8, 9). Especially, concerning suppression of alloimmunity, naturally occurring CD8^pos^ Tregs characterized as CD8^pos^CD122^pos^PD1^pos^ Tregs were shown to be more potent than conventional CD4^pos^ Tregs (10).

Both the CD8^pos^ Treg and Tresp pool contain CCR7^pos^CD45RA^pos^ naïve cells, which were shown to differentiate into CCR7^pos^CD45RA^neg^ central memory (CM), CCR7^neg^CD45RA^neg^ effector memory (EM), and CCR7^neg^CD45RA^pos^ terminally differentiated effector memory (TEMRA) cells. CM cells with various immune-stimulatory functions are considered as progenitor effector cells, whereas EM and TEMRA cells are thought to represent fully differentiated effector subsets (11). On the other hand, the naïve CD45RA^pos^ Treg/Tresp pool includes CD45RA^pos^CD31^pos^ recent thymic emigrant (RTE) cells, which can differentiate via CD45RA^neg^CD31^pos^ memory (CD31^pos^ memory) cells, via CD45RA^pos^CD31^neg^ mature naïve (MN) cells, or via direct proliferation into CD45RA^neg^CD31^neg^ memory (CD31^neg^ memory) cells. Thereby, MN cells seem to function as long-living reserve population in the naïve cell pool, preserving differentiation in case of RTE exhaustion (12). Combination of both differentiation approaches allows the identification of unexperienced CCR7^pos^CD45RA^pos^CD31^pos^ RTE cells (RTE Tregs/Tresps) and CCR7^pos^CD45RA^pos^CD31^neg^ MN cells (MN Tregs/Tresps), as well as CCR7^neg^CD45RA^pos^CD31^pos^ TEMRA cells (CD31^pos^ TEMRA Tregs/Tresps), and CCR7^neg^CD45RA^pos^CD31^neg^ TEMRA cells (CD31^neg^TEMRA Tregs/Tresps). The two TEMRA subsets might represent naïve T cells which have reached their maximum differentiation capacity at an earlier stage of differentiation.

Chronic kidney failure (KF) is known to impact the T cell compartment, leading to accelerated aging of distributed T cells (13–15) and subsequently reducing survival rates after kidney transplantation (16, 17). Recently, we demonstrated that chronic KF induces increased differentiation of naïve CD8^pos^ Tresps, resulting in the accumulation of CM, EM and TEMRA Tresps but a decrease in CM Tresp differentiation post-transplantation (18). CM Tresps are essential for effective immune stimulation and the early immune response. Accordingly, naïve CD8^pos^ Treg differentiation into EM Tregs was also proved to be strengthened in KF patients. After transplantation, immunosuppressive therapy prevented excessive differentiation of CD8^pos^ Tregs, but not CD8^pos^ Tresps. Therefore, exhaustion of CD8^pos^ Tresp differentiation may increase the risk of cancers such as non-melanoma skin cancer (NMSC), while preserving the differentiation capacity of CD8^pos^ Tregs, thus reducing the risk of graft rejection (19).

The objective of this study is to examine the differentiation of CD8^pos^ Tregs and Tresps in three distinct groups of kidney transplant recipients (KTR): those with stable allograft function, those who underwent an indication biopsy due to graft impairment, and those with stable function at enrolment but who developed future graft rejection. Our findings indicate an enhanced differentiation of RTE Tregs into MN Tregs and CM-enriched CD31^neg^ memory Tregs in patients who are likely to experience future rejection. In contrast, recipients experiencing rejection-related graft impairment showed an exhaustion of this differentiation, which was replaced by the proliferation of MN Tregs into EM-enriched CD31^neg^ memory Tregs. The resulting accumulation of CD31^neg^ TEMRA Tregs suggests a loss of proliferative capacity of RTE Tregs during rejection processes. Notably, no significant differences were found in the differentiation of CD8^pos^ Tresp cells.

## Material and methods

### Study participants

Blood samples were collected at the Department of Nephrology, University of Heidelberg, Germany. A total of 139 stable KTR with no signs of rejection and 41 KTR diagnosed with biopsy-confirmed acute renal graft rejection at the time of enrolment were sampled. Participants had undergone a kidney transplant at least three months prior to enrolment and had no recent illness or autoimmune disease. Exclusion criteria for stable KTR were suspected inflammation, suspected deterioration of graft function (serum creatinine > 2 mg/dl), or a history of malignancy. The group of participants with acute biopsy-confirmed graft rejection included patients with both T-cell-mediated and antibody-mediated rejection. Allograft biopsies at the time of blood collection were evaluated and diagnosed by the Institute of Pathology of the University of Heidelberg using semi-quantitative histological scores. Clinical acute rejection was defined as a rejection episode associated with graft dysfunction based on a greater than 30% increase in serum creatinine from baseline values and confirmed by pathological analysis of the biopsies according to the updated Banff classification.

We then followed the occurrence of acute renal graft rejection in the healthy group at a median of 2.1 (0.5 - 2.7) years after enrolment. Five patients developed acute renal graft rejection during this period. **Table 1** shows the clinical data of all participants at enrolment. The remaining 134 stable KTR did not develop acute renal graft rejection during this period.

**Table 1:** Clinical characteristics of the study participants.

	KTR with stable allograft	KTR with indication biopsy*	KTR with stable allograft developing rejection
	n = 134	n = 41	n = 5
Sex (female)	57 (43)	14 (34)	1 (20)
Age (y)	52 (23-82)	51 (18-78)	35 (26-55)
Time on dialysis (y)	4 (0-13)	4 (0-14)	1 (0-11)
Deceased donor kidney	77 (58)	22 (54)	3 (60)
Δ measuring time ** – date of transplantation (y)	7 (0-28)	4 (0-27)	3 (0-11)
Initial immunosuppression			
Tac + MPA + Steroid	50 (37)	19 (46)	1 (20)
CsA + MPA + Steroid	52 (39)	10 (24)	3 (60)
mTOR-inh. + MPA + Steroid	6 (4)	1 (3)	1 (20)
Azathioprine + others	4 (3)	1 (3)	0 (0)
Belatacept + others	2 (2)	0 (0)	0 (0)
Others	20 (15)	10 (24)	0 (0)
Biopsy result according to BANFF 2017			
II	1.3 (0.6-1.9)	6 (15)	2 (40)
III		30 (73)	1 (20)
IV		5 (12)	2 (40)
Serum creatinine (mg/dl) ***		2.6 (0.8-6.7)	1.5 (1.2-1.9)
CKD-EPI GFR (ml/min/1.73m²)***	61 (32-119)	24 (9-77)	48 (38-75)
C-reactive protein (mg/l) ***	neg (neg-15)	5 (neg-86)	1 (neg-3)

Categorial variables are presented as number (percentage), continuous variables as median (minimum-maximum).

CKD-EPI GFR, chronic kidney disease epidemiology collaboration estimated glomerular filtration rate; CsA, ciclosporin A; KTR, kidney transplant recipients; MPA, mycophenolic acid; mTOR-inh., mechanistic target of rapamycin-inhibitor; Tac, tacrolimus.

*indication biopsy due to deterioration of graft function.

**measures were performed 2018-2024.

***at measuring time.

The Regional Ethics Committee approved the study (reference number S-523/2012, 05.07.2018). All participants were fully informed about the study settings and aims. Written informed consent was received from all individuals.

### Fluorescence-activated cell staining

Nine ml of venous blood was collected into ethylenediaminetetraacetic acid (EDTA)-containing tubes. Peripheral blood mononuclear cells (PBMCs) were then isolated by density gradient centrifugation using Lymphodex (Inno-Train Diagnostik GmbH, Kronberg, Germany). PBMCs (8 x 10^6^) were then surface stained with fluorochrome conjugated undiluted monoclonal antibodies, directed against CD8, CD127, CCR7, CD45RA and CD31 (for further details, see **Supplementary Table S1**). After 20 minutes, PBMCs were washed twice with 3 ml phosphate-buffered saline (PBS) and centrifuged at 483 g for 5 minutes. Intracellular FoxP3 was detected using an anti-human FoxP3 staining kit (clone PCH101, eBioscience, Frankfurt, Germany), according to the manufacturer’s instructions. Negative control samples were incubated with isotype-matched antibodies. Cells were analyzed using a FACS Canto cytometer (BD Biosciences). Dead cells and doublets were excluded using forward and side scatter characteristics (FSC and SSC). Statistical analysis was based on a cell count of 100.000 CD8^pos^ T cells for each patient using an appropriate stopping gate.

### Gating strategy, cell subsets, and differentiation pathways

**Supplementary Figure S1** shows the gating strategy for these measurements. We determined the percentage of CD8^pos^ T cells of all lymphocytes and separated them into CD8^pos^CD127^low pos/neg^ FoxP3^pos^ Tregs and CD8^pos^CD127^pos^FoxP3^neg^ Tresps (**Supplementary Figures S1A–C**). We then subdivided both CD8^pos^ Tregs and Tresps into CCR7^pos^ CD45RA^pos^ naïve, CCR7^pos^ CD45RA^neg^ CM, CCR7^neg^ CD45RA^neg^ EM, and CCR7^neg^ CD45RA^pos^ TEMRA Tregs/Tresps (**Supplementary Figures S1D, F**) and in CD45RA^pos^CD31^pos^ RTE, CD45RA^pos^CD31^neg^ resting MN, CD45RA^neg^CD31^pos^ memory and CD45RA^neg^CD31^neg^ memory Tregs/Tresps (**Supplementary Figures S1E, G**). Finally, we merged these two differentiation schemes to distinguish newly released antigen-unexperienced CCR7^pos^CD45RA^pos^CD31^pos^ RTE cells (RTEs) from CCR7^neg^CD45RA^pos^CD31^pos^ TEMRA cells (CD31^pos^ TEMRAs) and CCR7^pos^CD45RA^pos^CD31^neg^ resting MN cells (resting MNs) from CCR7^neg^CD45RA^pos^CD31^neg^ TEMRA cells (CD31^neg^ TEMRAs).

This allowed us to identify three distinct differentiation pathways of CD8^+^ RTE T cells. First, they can differentiate into CD31^neg^ memory cells via CD31^pos^ memory cells (pathway 1) and could produce CD31^pos^ TEMRA cells when this pathway is exhausted. Second, RTE T cells can proliferate directly into CD31^neg^ memory cells (pathway 2) and could generate CD31^neg^ TEMRA cells when this proliferation is exhausted. Thirdly, RTE T cells can proliferate into resting MN cells, which may subsequently convert into CD31^neg^ memory cells or proliferate into CD31^neg^ memory cells (pathway 3). It appears that CM cells arise predominantly via pathway 1, whereas EM cells are more likely to arise via pathway 2. Resting MN cells may be able to convert or proliferate into CD31^neg^ memory cells, producing both CM and EM cells.

### Characterization of CD8^pos^FoxP3^pos^CD127^low pos/neg^ Tregs as CD8^pos^CD25^pos^CD127^low pos/neg^ Tregs

To characterize CD8^pos^FoxP3^pos^CD127^low pos/neg^ Tregs as CD8^pos^CD25^pos^CD127^low pos/neg^ Tregs or CD8^pos^FoxP3^pos^CD25^pos^ Tregs nine ml of venous blood was collected into ethylenediaminetetraacetic acid (EDTA)-containing tubes. Peripheral blood mononuclear cells (PBMCs) were then isolated by density gradient centrifugation using Lymphodex (Inno-Train Diagnostik GmbH, Kronberg, Germany). PBMCs (8 x 10^6^) were then surface stained with fluorochrome conjugated undiluted monoclonal antibodies, directed against CD8, CD127, CD25, and CD45RA (for further details, see **Supplementary Table S1**). After 20 minutes, PBMCs were washed twice with 3 ml phosphate-buffered saline (PBS) and centrifuged at 483 g for 5 minutes. Intracellular FoxP3 was detected using an anti-human FoxP3 staining set (clone PCH101, eBioscience) according to the manufacturer’s instructions. Negative control samples were incubated with isotype-matched antibodies. Cells were analyzed using a FACS Canto cytometer (BD Biosciences). Dead cells and doublets were excluded using forward and side scatter characteristics (FSC and SSC). Statistical analysis was based on a cell count of 100.000 CD8^pos^ Tcells using an appropriate stopping gate.

### Isolation and functionality test of CD8^+^CD25^+^CD127^low pos/neg^ Treg cells

For functional analysis of CD8^pos^CD25^pos^CD127^low pos/neg^ Tregs, we first isolated CD8^pos^ T cells by Magnetic-Associated Cell Sorting (MACS). An average of 75 ml of venous blood was collected in EDTA-containing tubes from 3 different healthy control subjects. Approximately, 13.2 x 10^7^ PBMCs per subject were obtained by density gradient centrifugation using Lymphodex (Inno-Train Diagnostik GmbH, Kronberg, Germany). The CD8^pos^ T cells were purified using the CD8^pos^ T cell Isolation Kit human (Miltenyi Biotec, Bergisch Gladbach, Germany), according to the manufacturer’s instructions. On average, 2.1 x 10^7^ CD8^pos^ T cells were isolated per subject. CD8^pos^ T cells were then surface stained with fluorochrome conjugated undiluted monoclonal antibodies, directed against CD8, CD127 and CD25 (for further details, see **Supplementary Table S1**). After 20 minutes, PBMCs were washed twice with 3 ml phosphate-buffered saline (PBS) and centrifuged with 483 g for 5 minutes. Afterwards, CD8^pos^ Tregs were isolated by cell sorting using a FACS Aria II cell sorter (BD Bioscience, Heidelberg, Germany). In all experiments, dead cells and doublets were excluded by FSC-A versus FSC-H gating, while the remaining cells were sorted into CD8^pos^CD25^pos^CD127^low pos/neg^ Tregs and the remaining CD8^pos^ Tresps. An average of 61.000 CD8^pos^CD25^pos^CD127^low pos/neg^ Tregs were received per subject.

To analyze the suppressive activity of the isolated CD8^pos^ Tregs, 2 × 10^4^ CD8^pos^ Tresps were co-cultured with the purified CD8^pos^CD25^pos^CD127^low pos/neg^ Tregs in a ratio of 1:1 to 1:128 in 96-well v-bottom plates. These suppression assays were performed in a final volume of 100 μl/well of X-VIVO15 medium (Lonza, Verviers, Belgium). For T-cell stimulation, the medium was supplemented with 1 μg/ml anti-CD3 (eBioscience, Frankfurt, Germany). As controls, CD8^pos^CD25^pos^CD127^low pos/neg^ Tregs and CD8^pos^ Tresps alone were cultured both with and without any stimulus. Cells were incubated at 37 °C in 5% CO_2_. After 4 days, 1 μCi ^3^H-thymidine (Hartmann Analytic, Braunschweig, Germany) was added to the cultures and the cells were incubated for a further 16 h. The cells were then harvested and ^3^H incorporation was measured by scintillation counting. The maximum suppressive activity (ratio of Tregs to Tresps 1:1 or 1:2) and the minimum ratio of Tregs to Tresps at which at least 15% suppression could be achieved were calculated.

### Statistical analysis

We used linear regression to examine changes in the percentage of CD8^pos^ T cells, Tregs, Tresps, and their subsets over the course of life with separate models for each patient group. Age-independent group differences were examined using multiple regression analysis adjusted for the age variable (centered on the mean), including an interaction term of the age and the patient group. A p-value < 0.05 was considered significant. However, this research is an exploratory study in which the calculated p-values are descriptive, but not confirmatory. BiAS for Windows (version 10.06) was used for all statistical tests.

## Results

### Kidney transplant recipients with future graft rejection show increased differentiation of RTE Tregs into CM Tregs

**Figure 1:**
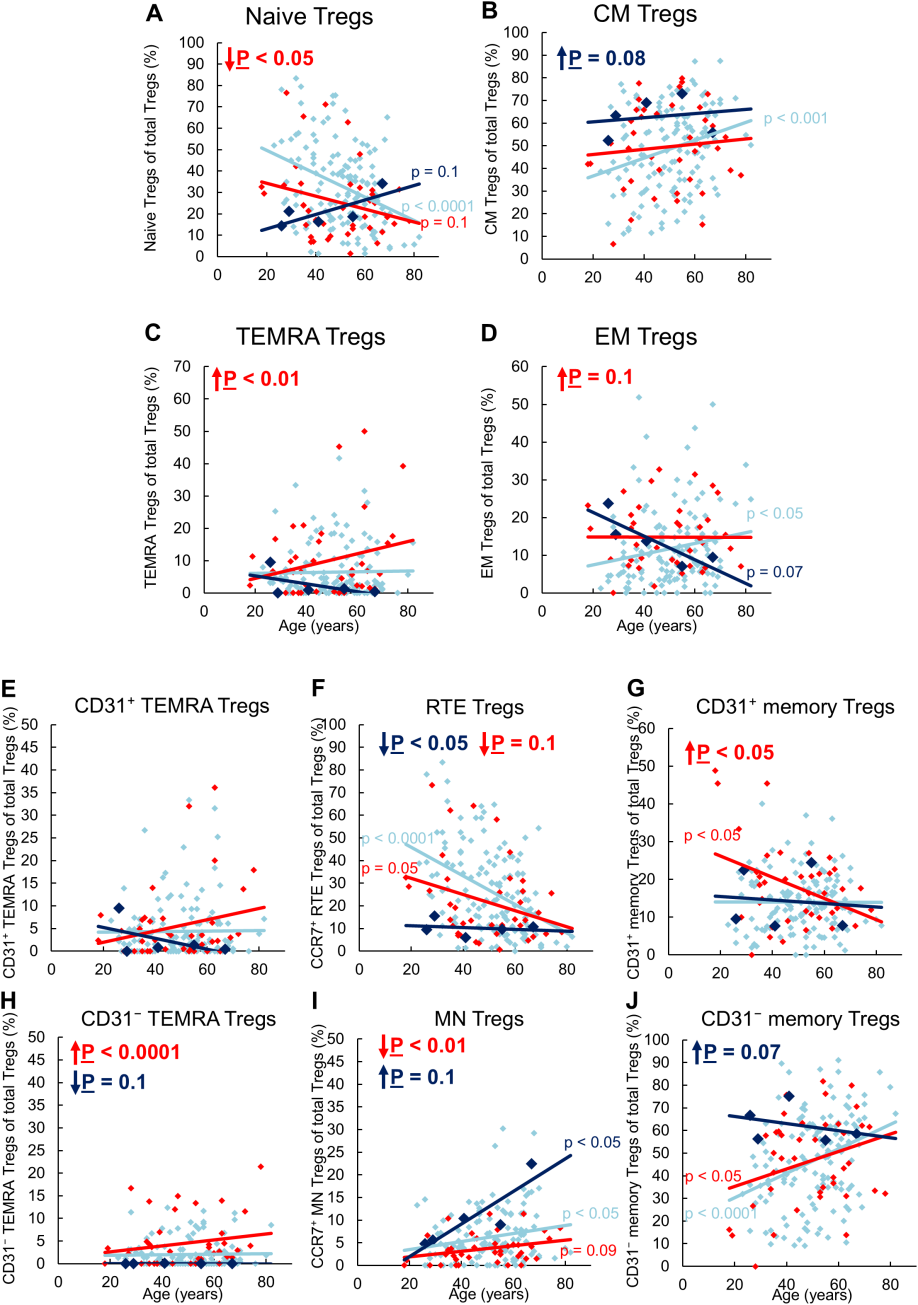
Differentiation of CD8^pos^ Tregs in KTR with stable allograft (n = 134), KTR with biopsy-proven rejection at enrolment (n = 41), and KTR who developed rejection during the follow-up period (n = 5). The figures show the percentage of naïve **(A)**, CM **(B)**, TEMRA **(C)**, and EM **(D)** Tregs within total Tregs of stable KTR in light blue (♦), KTR with rejection at enrolment in red (♦), and KTR who develop rejection during the follow-up period in dark blue (♦). To recognize different differentiation pathways, the figure also shows the proportion of CD31^pos^ TEMRA **(E)**, RTE **(F)**, CD31^pos^ memory **(G)**, CD31^neg^ TEMRA **(H)**, MN **(I)**, and CD31^neg^ memory Tregs **(J)** within total Tregs. Color-matched regression lines indicate changes with age, with significant changes indicated by the p-value next to the regression line. Age-independent significant differences of KTR with rejection at enrolment or KTR who develop rejection during the follow-up period compared to stable KTR are marked by an arrow and their color-matched P-values.

**Figure 1** shows the CD8^pos^ Treg cell differentiation of 134 stable KTR with stable allograft function, 5 KTR who developed allograft rejection during the follow-up period (up to a maximum of 3 years), and 41 patients with impaired graft function and consecutive indication biopsy, confirming graft rejection at the time of enrolment.

**Figure 2:**
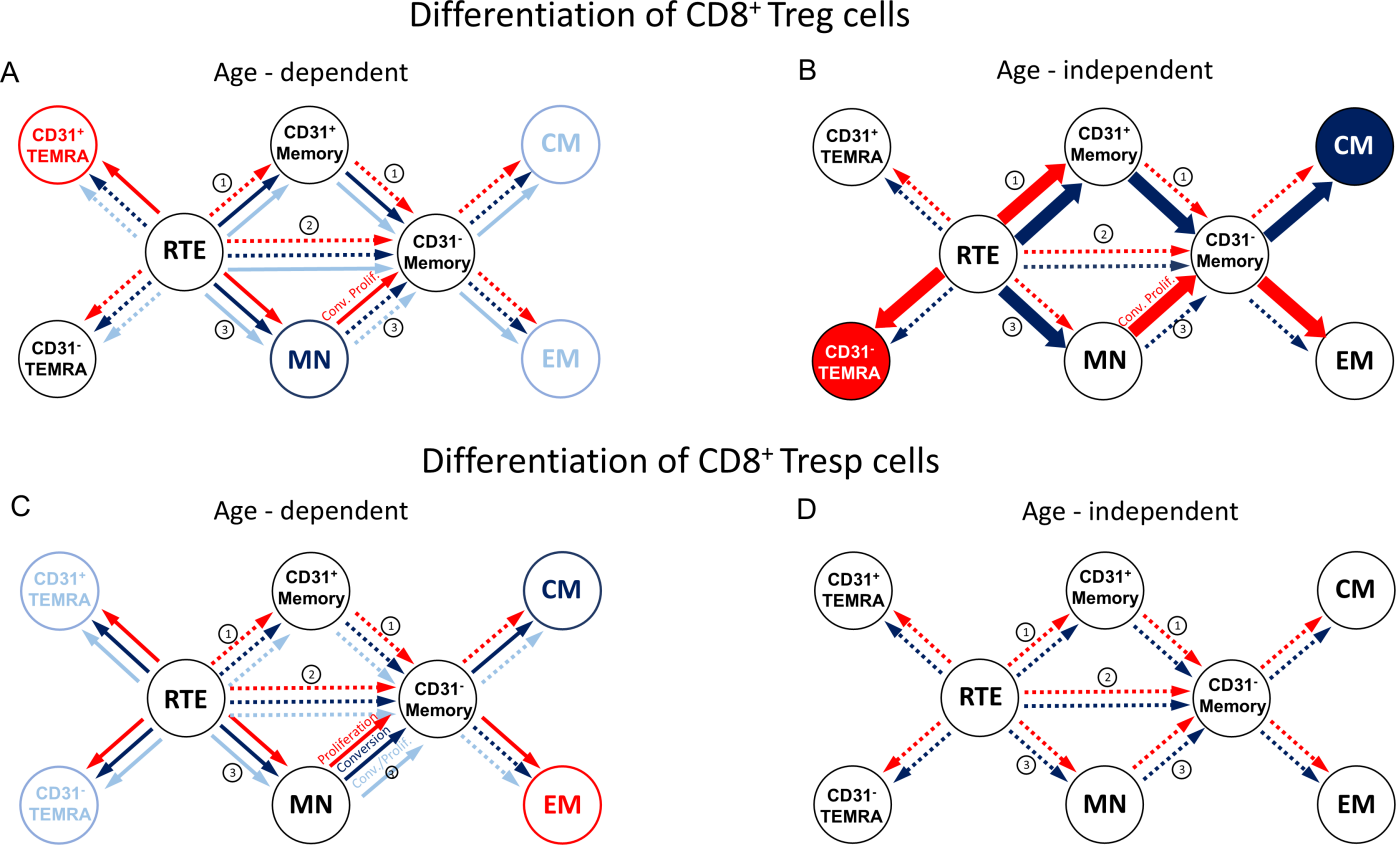
Proposed age-dependent **(A, C)** and age-independent enhanced **(B, D)** differentiation pathways of CD8^pos^ Tregs/Tresps for KTR with stable allograft function (light blue), KTR with rejection at enrolment in (red), and KTR who develop rejection during the follow-up period (dark blue). CD8^pos^ RTE T cells can differentiate via CD31^pos^ memory cells (1), directly proliferate (2), or differentiate via MN cells (3) into CD31^neg^ memory cells. Dotted arrows show possible differentiation, solid arrows show the age-dependent differentiation in KTR with stable allograft function (light blue), KTR with rejection at enrolment (red), and KTR with rejection during the follow-up period (dark blue). Bold arrows show the age-independently increased differentiation for KTR with rejection at enrolment (red) and of KTR who develop rejection during the follow-up period (dark blue) compared to KTR with stable allograft function.

In stable KTR, the percentage of naïve Tregs within the total Treg pool decreased with age, whereas the percentage of CM and EM Tregs increased significantly (**Figures 1A, B, D**). In contrast, the percentage of TEMRA Tregs remained unchanged (**Figure 1C**). Further subdivision of the naive Treg pool into RTE Tregs, MN Tregs, CD31^pos^ and CD31^neg^ TEMRA Tregs showed a significant decrease in RTE Tregs (**Figure 1F**), but an increase in resting MN and CD31^neg^ memory Tregs (**Figures 1I, J**), whereas both CD31^pos^ and CD31^neg^ TEMRA Tregs as well as CD31^pos^ memory Tregs did not change (**Figures 1E, G, H**). Therefore, our results suggest that in KTR with stable allograft function, RTE Treg differentiate via CD31^pos^ memory Tregs (pathway 1) or proliferate into CD31^neg^ memory Tregs (pathway 2). Thus, both CM and EM Tregs are increasingly produced with age, with resting MN Tregs also being enriched, presumably as a naive reserve population (**Figure 2A**).

Compared to stable KTR, those who developed a rejection during the follow-up period showed a trend towards an increased proportion of CM Tregs within the total Treg pool independent of age (**Figure 1B**). This is probably due to an increased differentiation of RTE Tregs via CD31^pos^ memory Tregs into CD31^neg^ memory Tregs (**Figure 2B**, pathway 1), as RTE Tregs are reduced, independently of age, whereas CD31^neg^ memory Tregs and resting MN Tregs are almost significantly increased (**Figures 1F, I, J**). A reduction in TEMRA Tregs, particularly those expressing CD31 (**Figure 1H**), may suggest enhanced proliferation of RTE Tregs (pathway 2) in EM Tregs, which appears to be markedly age-dependent (**Figure 1D**). However, due to the small number of patients in this group, significance was not reached. With age, naive Tregs tended to increase, TEMRA Tregs and CM Tregs remained relatively constant, while EM Tregs decreased almost significantly (**Figures 1A-D**). Further analysis of age-dependent changes within the total Treg pool showed a tendency for CD31^pos^ TEMRA Tregs as well as CD31^neg^ memory Tregs to decrease (**Figures 1E, J**), while all other subsets remained constant (**Figures 1F-H**), except for resting MN Tregs, which increased significantly with age (**Figure 1I**). Apparently, in these patients, RTE Treg differentiation via CD31^pos^ memory Tregs into CM Tregs (pathway 1) is preserved with age, rather than their proliferation into EM Tregs (pathway 2), with CD31^neg^ memory Tregs likely to be less enriched than MN Tregs (**Figure 2A**).

### Patients with biopsy-proven rejection at the time of enrolment show increased differentiation into TEMRA Tregs

In KTR with biopsy-proven rejection at the time of enrolment, the percentage of naïve Tregs in the total Treg pool was significantly reduced regardless of age, while that of TEMRA Tregs was significantly increased (**Figures 1A, C**). The percentage of EM Tregs also tended to be increased (**Figure 1D**) whereas that of CM Tregs remained unchanged compared to stable KTR (**Figure 1B**). Further subdivision of the naive Treg pool into RTE Tregs, MN Tregs, CD31^pos^ and CD31^neg^ TEMRA Tregs showed an almost significant decrease of RTE Tregs and a significant decrease of MN Tregs (**Figures 1F, I**), accompanied with a significant increase in CD31^pos^ memory Tregs and CD31^neg^ TEMRA Tregs (**Figures 1G, H**), while CD31^pos^ TEMRA Tregs and CD31^neg^ memory Tregs remained unchanged within the total Tregs (**Figures 1E, J**). Therefore, it seems that the exhaustion of RTE Treg differentiation via CD31^pos^ memory Tregs (pathway 1) and via their direct proliferation (pathway 2) was compensated by an increased differentiation of resting MN Tregs into CD31^neg^ memory Tregs, probably more by proliferation in EM Tregs than by conversion in CM Tregs (pathway 3), (**Figure 2B**). Thus, the proportion of CM Tregs within the total Treg pool was maintained, whereas that of EM Tregs was rather increased compared to stable KTR (**Figures 1B, D**). Compared to KTR with future rejection, CM Tregs decreased in KTR with current rejection (**Figure 1B**), also suggesting that resting MN Tregs are more likely to proliferate into CD31^neg^ memory Tregs (**Figure 2B**).

With age, these patients seemed to accumulate CD31^pos^ TEMRA Tregs and produced significantly fewer CD31^pos^ memory Tregs (**Figures 1E, G**), suggesting that RTE Tregs lose their ability to differentiate into CD31^neg^ memory Tregs via pathway 1. Furthermore, RTE Tregs decreased with age, whereas resting MN Tregs and CD31^neg^ memory Tregs increased almost significantly (**Figures 1F, I, J**). Therefore, it appears that only the ongoing differentiation of resting MN Tregs replenishes the CD31^neg^ memory Treg pool (**Figure 2A**). However, this means that the alternative differentiation of resting MN Tregs (pathway 3) in these patients cannot generate CM and EM Tregs as effectively with age as in stable KTR (**Figures 1B, D**).

### Neither kidney transplant recipients with future, nor those with current biopsy-proven rejection show differences in the differentiation of CD8^pos^ Tresp cells

**Figure 3:**
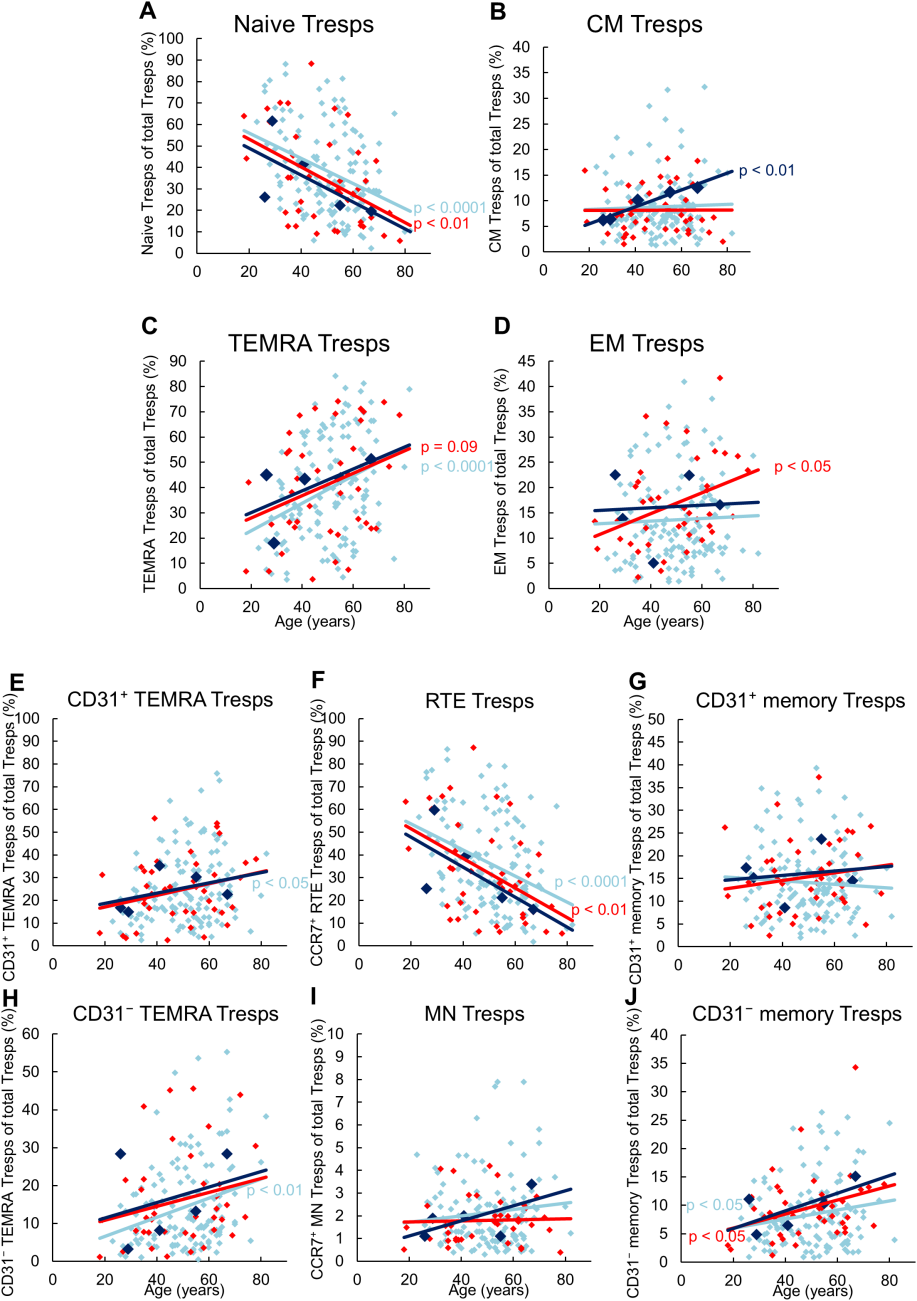
Differentiation of CD8^pos^ Tresps in KTR with stable allograft (n = 134), KTR with biopsy proven rejection at enrolment (n = 41), and KTR who developed rejection during the follow-up period (n = 5). The figures present the percentage of naïve **(A)**, CM **(B)**, TEMRA **(C)**, and EM **(D)** Tresps within total Tresps of stable KTR in light blue (♦), KTR with rejection at enrolment in red (♦), and KTR who develop rejection during the follow-up period in dark blue (♦). To recognize different differentiation pathways, the figure additionally shows the proportion of CD31^pos^ TEMRA **(E)**, RTE **(F)**, CD31^pos^ memory **(G)**, CD31^neg^ TEMRA **(H)**, MN **(I)**, and CD31^neg^ memory Tresps **(J)** within total Tresps. Color-matched regression lines indicate changes with age, with significant changes indicated by the p-value next to the regression line. Age-independent significant differences of KTR with rejection at enrolment or KTR who develop rejection during the follow-up period compared to stable KTR were not observed.

**Figure 3** shows the differences in CD8^+^ Tresp cell differentiation between the three patient groups. In stable KTR, there appeared to be an increased differentiation of naïve Tresps into TEMRA Tresps with age, as naïve Tresps decreased and TEMRA Tresps increased significantly, whereas CM and EM Tresps were maintained but did not increase significantly (**Figures 3A-D**). As both CD31^pos^ and CD31^neg^ TEMRA Tresps increased significantly with age (**Figures 3E, H**), pathways 1 and 2 appeared to be exhausted with age, so that RTE Tresps already differentiate via resting MNs into CD31^neg^ memory Tresps (pathway 3), replenishing the CM and EM Tresp pool (**Figure 2C**).

Compared to stable KTR, neither those who developed future rejection nor those who were diagnosed with current rejection showed age-independent differences in the differentiation of CD8^pos^ Tresps (**Figures 3A-J**, **2D**). Only with age, patients with future rejection showed an increasing differentiation of RTE Tresps into CM Tresps (**Figure 3B**), presumably due to increased conversion of resting MN Tresps into CD31^neg^ memory Tresps (**Figure 2C**), whereas KTR with current rejection showed an increasing differentiation into EM Tresps (**Figure 3D**), presumably due to increased proliferation of resting MN Tresps into CD31^neg^ memory Tresps (**Figure 2C**).

### Kidney transplant recipients who develop future graft rejection have an increased CD8^pos^ Treg/Tresp ratio

**Figure 4:**
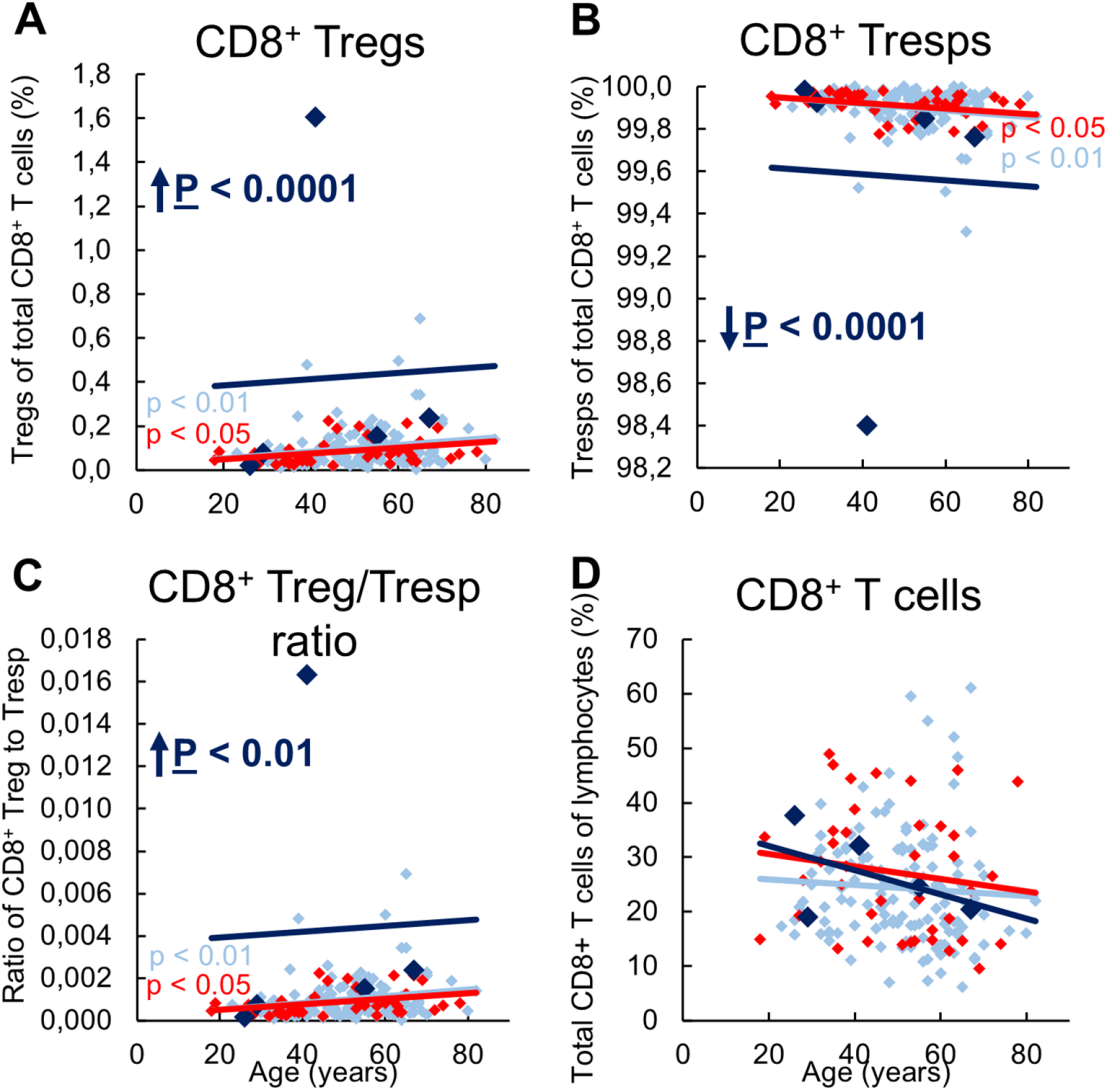
Composition of CD8^pos^ T cells with CD8^pos^CD127^low pos/neg^FoxP3^pos^ Tregs and CD8^pos^CD127^pos^FoxP3^neg^ Tresps in KTR with stable allograft function (n = 134), KTR with biopsy-proven rejection at enrolment (n = 41), and KTR who developed rejection during the follow-up period (n = 5) and its influence on their Treg/Tresp ratio and the percentage of total CD8^pos^ T cells. The diagrams exhibit the proportion of Tregs **(A)** and Tresps **(B)** within total CD8^pos^ T cells (cell count of 100.000 for each patient), the CD8^pos^ Treg/Tresp ratio **(C)**, and the percentage of total CD8^pos^ T cells of all lymphocytes **(D)** of KTR with stable allograft function in light blue (♦), KTR with biopsy-proven rejection at enrolment in red (♦), and KTR who developed rejection during the follow-up period in dark blue (♦). Color-matched regression lines indicate changes with age, with significant changes indicated by the p-value next to the regression line. Age-independent significant differences of KTR with rejection at enrolment or KTR who develop rejection during the follow-up period compared to stable KTR are marked by an arrow and their color-matched P-values.

We then investigated whether these differences in the differentiation of CD8^pos^ Tregs between the three patient groups had an impact on the composition of the total CD8^pos^ T cell pool with CD8^pos^ Tregs and CD8^+^ Tresps, their ratio to each other, and the proportion of total CD8^+^ T cells in all lymphocytes. In stable KTR, there was a significant increase in CD8^+^ Tregs with a decrease in Tresps and thus an increasing CD8^+^ Treg/Tresp ratio with age (**Figures 4A-C**), presumably due to an age-related stronger production of CD31^neg^ memory Tregs (**Figure 1J**) than CD31^neg^ memory Tresps (**Figure 3J**). In KTR with biopsy-proven rejection the same age-related changes were found, presumably because in these patients, the age-dependent differentiation that normally occurs in healthy individuals can be adequately replaced by differentiation of resting MN Tregs/Tresps.

Regardless of age, KTR developing future rejection showed a significantly increased percentage of CD8^pos^ Tregs and a complementary decreased percentage of CD8^pos^ Tresps, resulting in an increased Treg/Tresp ratio compared to stable KTR (**Figures 4A-C**). The percentage of total CD8^pos^ T cells did not change between the three groups (**Figure 4D**), indicating that there was indeed an increase in Tregs but decrease in Tresps.

### Purified CD8^pos^CD25^pos^CD127^low pos/neg^ Tregs show suppressive activity

**Figure 5:**
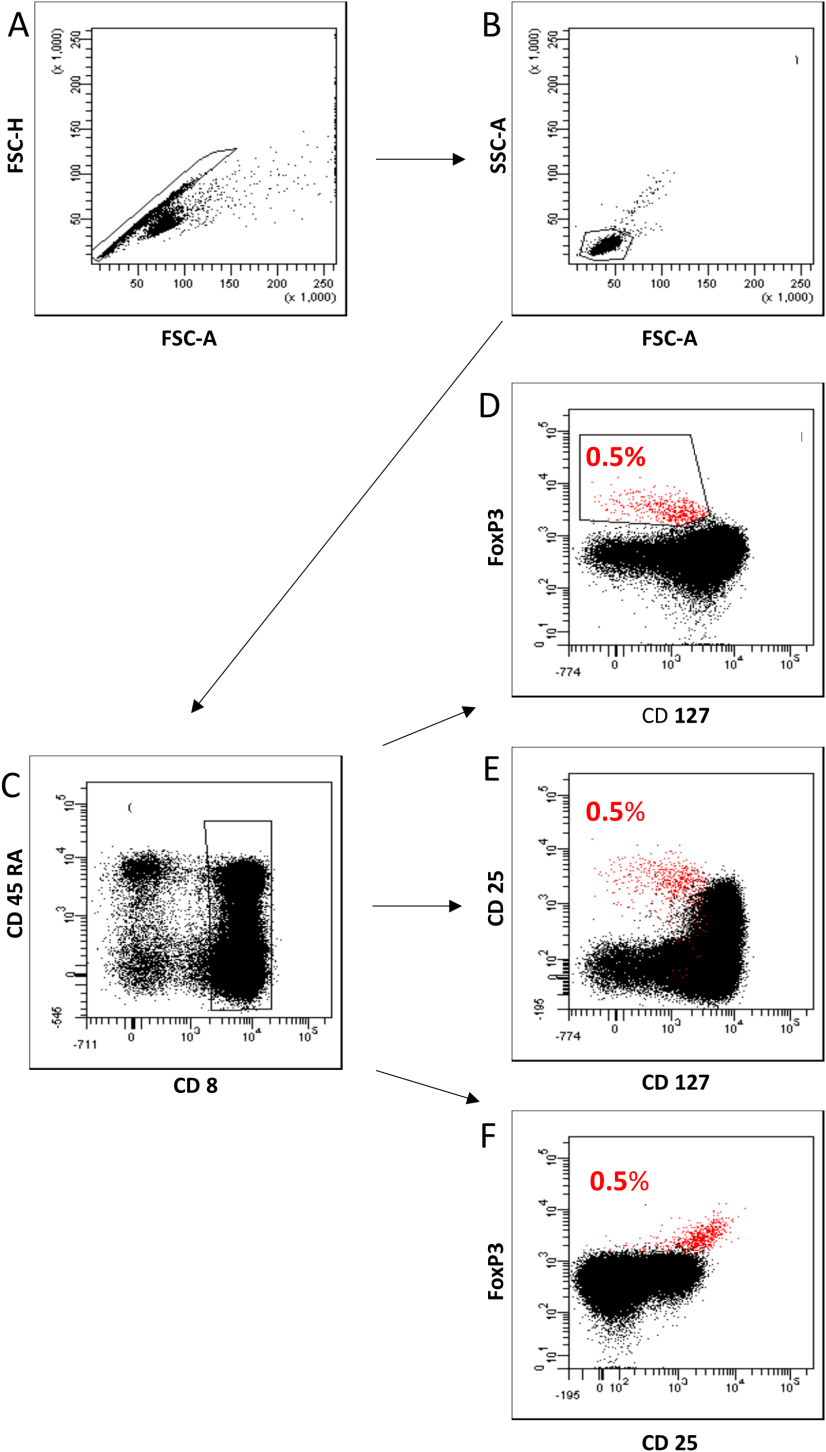
Characterization of CD8^pos^FoxP3^pos^CD127^low pos/neg^ Tregs as CD8^pos^CD25^pos^CD127^low pos/neg^ Tregs and CD8^pos^FoxP3^pos^CD25^pos^ Tregs. First, all lymphocytes were detected by FSC-H versus FSC-A **(A)** and subsequent FSC-A versus SSC-A **(B)** gating. Then, we examined the fluorescence activity of CD8 versus CD45RA to detect CD8^pos^ T cells within all lymphocytes **(C)**. Fluorescence activity of FoxP3 versus CD127 was presented to separate CD8^pos^ Tregs from Tresps. CD8^pos^ Tregs were gated as CD8^+^FoxP3^+^CD127^low pos/neg^ Tregs within total CD8^pos^ T cells (0.5%) **(D)** and presented as CD8^pos^CD25^pos^CD127^low pos/neg^ Tregs (97% overlap) **(E)** and CD8^pos^FoxP3^pos^CD25^pos^ Tregs (100% overlap) **(F)** by analyzing fluorescence activity of CD25 versus CD127 and FoxP3 versus CD25.

**Figure 6:**
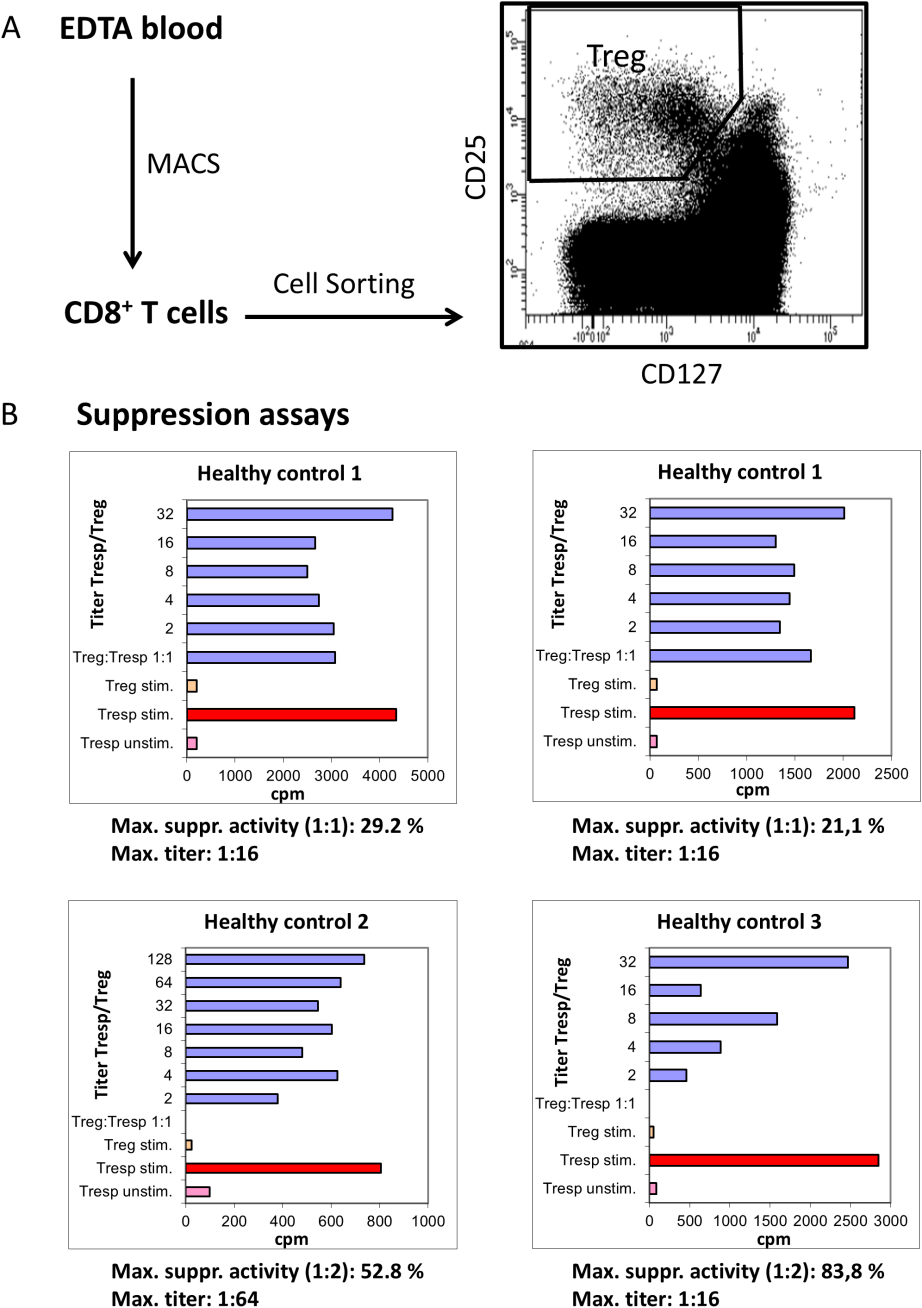
Suppressive activity of CD8^pos^CD25^pos^CD127^low pos/neg^ Tregs. For functional analysis, CD8^pos^ T cells were isolated by Magnetic-Associated Cell Sorting (MACS) from three different healthy controls. CD8^pos^ T cells were then sorted into CD8^pos^CD25^pos^CD127^low pos/neg^ Tregs and the remaining CD8^pos^ Tresps by analyzing fluorescence activity of CD25 versus CD127 using a cell sorter **(A)**. Using suppression assays in which the separated CD8^pos^CD25^pos^CD127^low pos/neg^ Tregs were co-cultured with Tresps, the suppressive activity of CD8^pos^CD25^pos^CD127^low pos/neg^ Tregs was estimated (cpm=counts per minute) in three different healthy controls (for healthy control 1 the reproducibility was estimated), by determining the maximum suppressive activity (Titer Tregs/Tresp 1:1 or 2:1) and the Titer of Tregs/Tresps up to which a minimum suppressive activity of 15% was achieved **(B)**. For T-cell stimulation, the medium was supplemented with 1 μg/ml anti-CD3 (eBioscience, Frankfurt, Germany). The control bars, Treg stim. (stimulated) and Tresp unstim. (unstimulated) showed no proliferation. The red coloured bars illustrate the stimulated Tresps without co-culture with Tregs.

To demonstrate that CD8^pos^FoxP3^pos^CD127^low pos/neg^ Tregs have suppressive capabilities, we used FACS analysis to show that these cells can also be reliably detected as CD8^pos^FoxP3^pos^CD25^pos^ Tregs or CD8^pos^CD25^pos^CD127^low pos/neg^ Tregs. In our experiments, we found an overlap of 100% for CD8^pos^FoxP3^pos^CD25^pos^ Tregs and 97.1% for CD8^pos^CD25^pos^CD127^low pos/neg^ Tregs with CD8^pos^FoxP3^pos^CD127^low pos/neg^ Tregs (**Figure 5**). These analyses demonstrate the possibility of detecting CD8^pos^ Tregs without detecting the FoxP3 marker, which is an intracellularly expressed transcription factor, that requires cell fixation for intracellular staining. Therefore, we first isolated CD8^pos^ T cells by MACS technology and then used cell sorting to separate CD8^pos^CD25^+^CD127^low pos/neg^ Tregs from CD8^pos^ Tresps (**Figure 6A**).

To demonstrate the suppressive activity of CD8^pos^CD25^pos^CD127l^ow pos/neg^ Tregs we used suppression assays, in which CD8^pos^CD25^pos^CD127^low pos/neg^ Tregs and CD8^pos^ Tresps were co-cultured at ratios of 1:1 - 1:128. Maximum suppressive activity was calculated at a Treg/Tresp ratio of 1:1 or 1:2. In addition, the maximum titer of CD8^pos^CD25^pos^CD127^low pos/neg^ Tregs was determined at which a minimum suppressive activity of 15% was achieved. **Figure 6B** shows these results for three healthy controls. These experiments were performed twice in one individual (healthy control 1) to ensure reproducibility. Our results show that CD8^pos^CD25^pos^CD127^low pos/neg^ Tregs from healthy control 1 suppressed the proliferation of CD8^pos^ Tresps with a maximum suppressive activity of 29.8% and 21.1%, respectively, while the other individuals reached 52.8% and 83.8%, respectively. The titer up to which a minimum suppressive activity of 15% could be achieved was 1:16 in both experiments for healthy control 1 and 1:64 and 1:16 for healthy controls 2 and 3, respectively (**Figure 6B**).

Summarizing, our results reveal that in patients at higher risk of future rejection, RTE Tregs show enhanced differentiation into MN Tregs and CM-enriched CD31^neg^ memory Tregs. Conversely, in recipients with rejection-related graft impairment, this differentiation process appears to be exhausted, instead favoring the proliferation of MN Tregs into EM-enriched CD31^neg^ memory Tregs. This leads to an accumulation of CD31^neg^ TEMRA Tregs, indicating a loss of the proliferative capacity of RTE Tregs during rejection events.

## Discussion

In healthy individuals, an age-dependent increase in the Treg/Tresp ratio of both CD4^pos^ and CD8^pos^ T cells provides protection against autoimmune diseases, but increases susceptibility to infection and cancer in the elderly (18, 20, 21). Not only their number, but also their function is ensured by special differentiation pathways that these cells adopt as they age (20). For CD4^pos^ T cells, our previous studies in patients with autoimmune disease have shown that the appropriate ratio of Treg/Tresp cells and their functionalities are maintained by altering the differentiation pathways of RTE Tregs/Tresps so that when one pathway is exhausted, another can be used. This ensures sufficient differentiation of RTE Tregs/Tresps into memory Tregs/Tresps, with the additional differentiation of resting MN Tregs/Tresps (pathway 3) being particularly important in maintaining both age-dependent and age-independent enhanced differentiation in specific diseases (20–22). For CD8^pos^ T cells, our recent studies in patients with chronic kidney failure revealed an exaggerated age-independent differentiation of CD8^pos^ RTE Tregs/Tresps via pathways 1 and 2, resulting in the enhanced production of EM Tregs/Tresps and, in particular, apoptosis-resistant CM Tresps with strong Fas ligand-mediated cytotoxicity. However, differentiation via pathway 1 could not be maintained in KTR, so that both CM Tregs and CM Tresps were severely depleted. Sufficient Treg/Tresp proliferation maintained EM Treg/Tresp production via pathway 2. Nevertheless, increased differentiation of TEMRA Tregs/Tresps was observed in KTR. In addition, we found, that thymic output, which has been shown to be reduced in patients with kidney failure (17), but restored after transplantation (23), allowed regular age-dependent differentiation of both Tregs and Tresps in KTR, whereas the immunosuppressive therapy successfully prevented excessive Treg differentiation, but not as sufficiently that of Tresps (18).

In the current study, we show an age-independent increased differentiation of RTE Tregs via pathway 1, resulting in abundant CM Treg production in KTR, who developed future rejection during the follow-up period. However, no age-related or age-independent differences were found in the differentiation of RTE Tresps in KTR who develop future graft rejection. These results suggest that in the context of a rejection process, a prolonged, chronically activated differentiation of functionally highly potent CM Tregs precedes the actual rejection. It appears that these cells, which can then prevent rejection, develop long before the deterioration of kidney function becomes apparent. However, as this differentiation cannot be maintained in the long term, Tresp-mediated rejection appears to occur when the differentiation of these CM Tregs exhausts. Therefore, an elevated Treg/Tresp ratio combined with an increased proportion of CM Tregs in unremarkable KTR suggests that these patients are at risk for future development of rejection. Thus, further studies may be needed to confirm this early accumulation of highly activated CM Tregs in KTR with future rejection, as these patients may require more frequent creatinine monitoring and presumably more intensive immunosuppressive therapy to prevent impending rejection.

Our data obtained in KTR with ongoing rejection at the time of enrolment show that differentiation was maintained by conversion and proliferation of resting MN Tregs. The increased percentage of both CD31^pos^ memory and CD31^neg^ TEMRA Tregs suggests insufficiencies in RTE Treg differentiation and proliferation (pathways 1 and 2). Obviously, the differentiation of resting MN Tregs, cannot maintain elevated levels of CM Tregs, ultimately causing the graft rejection. We show that CD31^neg^ TEMRA Tregs might represent a marker population in peripheral blood of KTR as they were highly significantly increased in patients with ongoing rejection. Our previous findings showing that age-related exhaustion of CM Tresp differentiation leads to non-melanoma skin cancer (NMSC), particularly in older KTR, show that similar differentiation mechanisms as those observed in CD8^pos^ Tresp cells in KTR with future or current NMSC can also occur in CD8^pos^ Treg cells, but then more likely in KTR with future or current rejection (19).

Our study has important limitations, (a) few patients developing future rejection, (b) the low number of CD8^pos^ Tregs, (c) the study period from 2018-2024 including kidney transplant recipients from 1990-2023 with both tacrolimus and ciclosporin as primary immunosuppression possibly influencing differentiation pathways concerning immunosuppressive capacity and (d) the analyses using a FACS Canto cytometer (BD Biosciences) were limited to 6 different colors. Therefore, we were not able to additionally stain the cells with antibodies directed against CD122 or CD4 which should be a future aim. Furthermore, it remains controversial whether the CD8^pos^ T cell population, i.e. CCR7^neg^ but co-expressing the CD45RA marker (TEMRA T cells), represent naive T cells or memory T cells that are re-expressing the CD45RA marker (24). These cells have now been shown to be heterogeneous, containing differentially differentiated T cells with different phenotypic and functional properties, dividing this population into a subset of terminally differentiated T cells (CD57^pos^) and a more naïve population with greater differentiation plasticity (CD57^neg^) (25). Further studies are needed to determine whether this also applies to CD31^pos^ TEMRA and CD31^neg^ TEMRA T cells.

Recent studies suggest that kidney allograft rejection may be influenced by the differentiation of certain CD8^pos^ effector T cells, such as CD28^neg^CD8^pos^ T cells (26), as well as other immune cells like B cells and subsets of CD4^pos^ T cells, some of which have regulatory roles. These findings indicate that an imbalance between effector and regulatory immune cells is a key factor in allograft rejection (27). However, the exact role of CD8^pos^ T cells in either rejection or acceptance of kidney allografts remains unclear. Recently, gene signatures of regulatory T cells, Th1, Th2, Th17 cells, T follicular helper cells, and CD4^pos^ and CD8^pos^ tissue-resident memory T cells were found to be enriched in biopsies from patients with T cell-mediated rejection (TCMR). Analysis of graft-infiltrating cells through gene expression patterns identified CD8^pos^ T cells as the most prevalent T cell subtype in allografts undergoing TCMR (28) or mixed antibody-mediated rejection (ABMR)/TCMR (29). Additionally, recent findings show that the immune cell composition in renal allografts does not align well with the primary rejection categories defined by the Banff criteria. Only the accumulation of CD8^pos^ TEMRA cells has demonstrated a strong and consistent link to graft failure, independent of the Banff rejection phenotype (30). The distinction between TCMR and ABMR does not capture the full immunopathological picture (31). Studies using multiplex immunofluorescence and immunohistochemistry on kidney transplant biopsies have provided evidence of the mismatch between Banff rejection categories and the actual composition of infiltrating immune cells (32, 33).

Previous research on the long-term effects of CD8^pos^ T cell differentiation supports the idea that monitoring CD8^pos^ T cell subsets could enhance early identification of patients at risk. Although initial studies did not distinguish between Tregs and Tresps, the expansion of CD8^pos^ TEMRA cells in KTR was found to be linked to long-term graft dysfunction over a 15-year follow-up period (34). Further studies revealed that a higher frequency of a subpopulation of CD8^+^ TEMRA T cells with potent cytotoxic activity can identify KTRs at high risk for graft failure (35). This suggests that the terminal differentiation of alloreactive CD8^pos^T cells plays a critical role. However, it remains uncertain whether this differentiation is essential for graft acceptance or rejection. In mouse models, transferring alloreactive CD8^pos^ T cells into T-cell-depleted syngeneic mice led to spontaneous long-term acceptance of liver grafts, while acute rejection occurred in kidney and heart grafts (36). These findings imply that clonal exhaustion or deletion of the alloreactive CD8^pos^ Tresp repertoire may induce tolerance spontaneously, while rejection and memory responses might also be affected by exhaustion of other cell types, such as CD4^pos^ and CD8^pos^ Treg cells. Several studies have shown a correlation between T cell exhaustion and better outcomes in kidney transplantation. For instance, the expansion of a circulating PD-1^pos^ CD57^neg^ subset in both CD4^pos^ and CD8^pos^ T cells has been linked to improved graft function in KTR (37), and a higher incidence of exhausted T cell subsets was associated with a decline in acute rejection post-transplantation (38). In contrast, administering anti-PD-1 antibodies was found to trigger acute graft rejection (39). It is also important to note that even highly cytotoxic CD8^pos^ TEMRA cells, which have distinct expression profiles, can experience exhaustion, as indicated by the co-expression of immune-inhibitory markers like PD-1, which suppress their functionality (40).

In the future, it will be crucial to investigate the differentiation of CD8^pos^ Treg and Tresp cells separately to identify which subpopulation’s exhaustive differentiation most significantly affects rejection processes. Our findings suggest that the exhaustive differentiation of CD8^pos^ CM Treg cells may play a more critical role in driving rejection. This exhaustion stems from increased differentiation into CM Tregs, which initially seems to prevent graft rejection. The potential involvement of CD8^pos^ Treg cells in transplantation has been well-established in both animal models and human studies (41). However, further research is necessary to pinpoint specific surface markers that can monitor their differentiation under various immunosuppressive therapies and clinical conditions for diagnostic and therapeutic purposes.

## Data Availability

The original contributions presented in the study are included in the article/**Supplementary Material**. Further inquiries can be directed to the corresponding author.

## Supplementary material

Supplementary Figure 1 Gating strategy for six-color-flow-cytometric division of CD8^pos^CD127^low pos/neg^FoxP3^pos^ Tregs and CD8^pos^CD127^low pos/neg^FoxP3^neg^ Tresps into their subsets. First, all lymphocytes (P1) were detected by side scatter characteristics (SSC) versus forward scatter characteristics (FSC) **(A)**. Then, we examined the fluorescence activity of CD8 versus CD45RA **(B)** to determine the percentage of CD8^pos^ T cells of all lymphocytes (P2). Afterwards, the fluorescence activity of FoxP3 versus CD127 was presented **(C)** to separate CD8^pos^ Tregs (P3) from Tresps (P4). Finally, Tregs and Tresps were separately divided into RTE (P6, P14), MN (P8, P16), CD31^pos^ memory (P5, P13), and CD31^neg^ memory cells (P7, P15) cells, respectively, by analyzing the fluorescence activity of CD45RA versus CD31 **(D, F)**. The percentage of naive (P10, P18), CM (P9, P17), EM (P11, P19) and TEMRA (P12, P20) Tregs/Tresps was identified by using fluorescence activity of CD45RA versus CCR7 **(E, G)**.

Supplementary Table 1 Illustration of the Fluorochromes used with the associated antigen and volume used for staining.
