# Supplementary figures and images for: Exhaustion of CD8pos central memory regulatory T cell differentiation is involved in renal allograft rejection
Source: Front Immunol. 2025 Jan 24;16:1532086. doi: 10.3389/fimmu.2025.1532086 (PMC11802571; doi:10.3389/fimmu.2025.1532086)

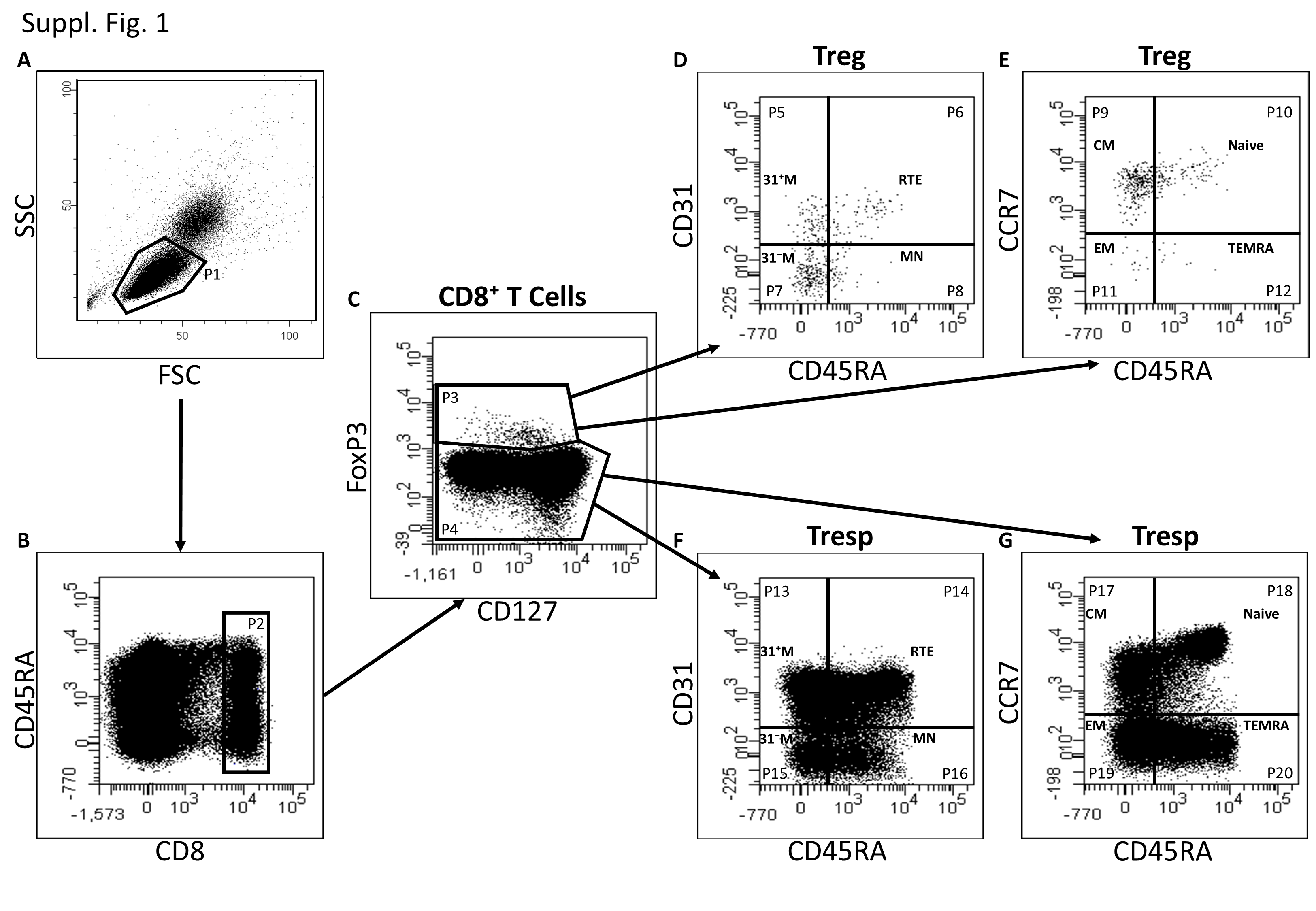

Supplement: Supplementary Figure 1 — Gating strategy for six-color-flow-cytometric division of CD8posCD127low pos/negFoxP3pos Tregs and CD8posCD127low pos/negFoxP3neg Tresps into their subsets. First, all lymphocytes (P1) were detected by side scatter characteristics (SSC) versus forward scatter characteristics (FSC) (A). Then, we examined the fluorescence activity of CD8 versus CD45RA (B) to determine the percentage of CD8pos T cells of all lymphocytes (P2). Afterwards, the fluorescence activity of FoxP3 versus CD127 was presented (C) to separate CD8pos Tregs (P3) from Tresps (P4). Finally, Tregs and Tresps were separately divided into RTE (P6, P14), MN (P8, P16), CD31pos memory (P5, P13), and CD31neg memory cells (P7, P15) cells, respectively, by analyzing the fluorescence activity of CD45RA versus CD31 (D, F). The percentage of naive (P10, P18), CM (P9, P17), EM (P11, P19) and TEMRA (P12, P20) Tregs/Tresps was identified by using fluorescence activity of CD45RA versus CCR7 (E, G). [file Image1.tif]
