# Supplementary material for: Exhaustion of CD8pos central memory regulatory T cell differentiation is involved in renal allograft rejection
Source: Front Immunol. 2025 Jan 24;16:1532086. doi: 10.3389/fimmu.2025.1532086 (PMC11802571; doi:10.3389/fimmu.2025.1532086)
Supplement: Supplementary Table 1 — Illustration of the Fluorochromes used with the associated antigen and volume used for staining. [file Table1.docx]

**Supplementary Table 1:** Fluorochrome details

| **Fluorochrome** | **Antigen** | **Volume** |
| --- | --- | --- |
| PerCP (peritinin -chlorophyll-protein)  (BD Biosciences, Heidelberg, Germany) | CD8 | 10 µl |
| PE-Cy7 (phycoerythrin-cyanine 7)  (eBiosciences, Frankfurt, Germany) | CD127 | 5 µl |
| PE (phycoerythrin)  (Biolegend, SanDiego, USA) | CCR7 | 5 µl |
| APC-H7 (allophycocyanine-H7)  (BD Biosciences, Heidelberg, Germany) | CD45RA | 5 µl |
| Alexa Fluor-647  (BD Biosciences, Heidelberg, Germany) | CD31 | 5 µl |
| PE (phycoerythrin)  (BD Biosciences, Heidelberg, Germany) | CD25 | 20 µl |
| FITC (fluorescein isothiocyanate)  (eBiosciences, Frankfurt, Germany) | FOXP3 (clone PCH101) | 5 µl |
